# Network connectivity predicts language processing in healthy adults

**DOI:** 10.1002/hbm.25042

**Published:** 2020-05-25

**Authors:** Dardo Tomasi, Nora D. Volkow

**Affiliations:** ^1^ National Institute on Alcohol Abuse and Alcoholism Bethesda Maryland USA; ^2^ National Institute on Drug Abuse Bethesda Maryland USA

**Keywords:** brain–behavior prediction, cognition, connectome‐based predictive modeling, default mode network, feature selection, language, motion, precuneus

## Abstract

Brain imaging has been used to predict language skills during development and neuropathology but its accuracy in predicting language performance in healthy adults has been poorly investigated. To address this shortcoming, we studied the ability to predict reading accuracy and single‐word comprehension scores from rest‐ and task‐based functional magnetic resonance imaging (fMRI) datasets of 424 healthy adults. Using connectome‐based predictive modeling, we identified functional brain networks with >400 edges that predicted language scores and were reproducible in independent data sets. To simplify these complex models we identified the overlapping edges derived from the three task‐fMRI sessions (language, working memory, and motor tasks), and found 12 edges for reading recognition and 11 edges for vocabulary comprehension that accounted for 20% of the variance of these scores, both in the training sample and in the independent sample. The overlapping edges predominantly emanated from language areas within the frontoparietal and default‐mode networks, with a strong precuneus prominence. These findings identify a small subset of edges that accounted for a significant fraction of the variance in language performance that might serve as neuromarkers for neuromodulation interventions to improve language performance or for presurgical planning to minimize language impairments.

## 1 INTRODUCTION

Language, a unique human ability, is supported by a distributed network of frontoparietal (FPN) cortices, including classical language areas in the inferior frontal cortex (Broca's area) and the posterior superior temporal cortex (Wernicke's area), as well as temporal regions of the default‐mode network (DMN), visual areas, and the basal ganglia (Tomasi & Volkow, 2012). Converging evidence from lesion and neuroimaging data suggests that intrinsic functional connectivity (iFC) among these regions influences language production or comprehension (Chai et al., 2016; Doucet et al., 2015; Vigneau et al., 2006). iFC neuromarkers of language processing could be useful for guiding neuromodulation interventions that use repetitive transcranial magnetic stimulation (rTMS) or transcranial direct current stimulation (tDCS) to treat patients with language disorders (Fridriksson et al., 2018; Naeser et al., 2005) or to improve language performance with aging (Rezaee & Dutta, 2020). They could also help with presurgical brain mapping to minimize adverse effects on language (Sair, Agarwal, & Pillai, 2017). Though there is evidence that models combining behavioral and neuroimaging data could serve as neuromarkers to help predict children at risk for poor reading skills (Hoeft et al., 2007), little is known about whether imaging data can be used to predict language skills in healthy adults.

Machine learning methods, such as connectome‐based predictive modeling (CPM) (Rosenberg et al., 2016; Shen et al., 2017), are being used to predict a wide range of human behaviors (Beaty et al., 2018; Bellucci, Hahn, Deshpande, & Krueger, 2019; Emerson et al., 2017; Fountain‐Zaragoza, Samimy, Rosenberg, & Prakash, 2019; Greene, Gao, Scheinost, & Constable, 2018; Lin et al., 2018; Liu, Liao, Xia, & He, 2018; Rosenberg et al., 2016; Scheinost et al., 2019) and symptoms (Lake et al., 2019; Lin et al., 2018; Yip, Scheinost, Potenza, & Carroll, 2019). The popularity of CPM relies on its simple methodology and its leave‐one‐out cross‐validation (LOOCV) approach, which provides some protection against overfitting. Briefly, at each of *n* iterations, CPM predicts behavioral data from one of the *n* subjects based on imaging and behavioral data from the remaining *n* − 1 subjects. The *n* − 1 subjects are used to: (a) select features (functional connections, or “edges”, that are sensitive to a particular behavior) from large connectivity matrices **M** with ∼10^4^ edges using correlation analysis; (b) compute two summary measures (e.g., *positive* and *negative network strengths*) that correlate with the behavior across *n* − 1 subjects; and (c) optimize the parameters of a linear regression model associating behavior with the summary measures. Then, the optimal linear model and features are used to predict the behavior of the subject left aside.

Prior studies showed that brain–behavior correlations are prone to a high rate of false positives (Vul, Harris, Winkielman, & Pashler, 2009), which could impact the CPM's *feature selection* (FS) step. Thus, the significance of the findings emerging from CPM studies based only on leave‐one‐out (LOO) internal validation (within the same sample) may not always be warranted. While, the inclusion of FS within the LOOCV loop (in contrast to exclusion of FS from the LOOCV loop) provides some protection against false positives (Shen et al., 2017), little is known about the differences in performance between FS inclusion versus FS exclusion as it relates to false positives in FS, prediction accuracy and reproducibility of brain–behavior associations using traditional twofold cross‐validation (in an independent sample).

The importance of cross‐validation for brain–behavior correlation analyses is recognized by many as fundamental (Greene et al., 2018; Scheinost et al., 2019; Shen et al., 2017) and while several studies have validated their results in independent samples (Beaty et al., 2018; Greene et al., 2018; Kumar et al., 2019; Nostro et al., 2018; Rosenberg et al., 2016; Yoo et al., 2018) others relied only on LOOCV (Bellucci et al., 2019; Finn et al., 2015; Hsu, Rosenberg, Scheinost, Constable, & Chun, 2018; Lin et al., 2018; Yip et al., 2019). It is claimed that LOOCV is at the core for improved generalizability of CPM results compared to simple brain–behavior correlation analyses (Shen et al., 2017). However, prediction errors are more variable for LOOCV than for other cross‐validation strategies (Hastie, Tibshirani, & Friedman, 2017; Kohavi, 1995) such as twofold cross‐validation, in which a set of *n*/2 subjects is set aside as a “test sample” to assess the performance of the prediction model developed with the remaining *n*/2 subjects used as a “training sample.” One of the goals for this study was to compare LOOCV and twofold cross‐validation in the prediction of language scores from functional magnetic resonance imaging (fMRI) data as a function of sample sizes and of task or rest conditions. We hypothesized that regardless of sample size, FS inclusion within the LOOCV loop would decrease false positives compared with FS exclusion from the LOOCV loop, and that for large samples brain behavior predictions would not differ between LOOCV and twofold cross‐validation but that for small samples (*n* < 100) cross‐validation would minimize false positives. Based on prior findings (Greene et al., 2018; Jiang et al., 2020), we also hypothesized that connectivity maps derived from task‐fMRI sessions would improve the prediction of language scores, compared to those from rest‐fMRI sessions.

An additional goal for our study was to quantify the effect of motion in the prediction of language from fMRI data since spurious connectivity patterns can arise from head movements (Birn, Diamond, Smith, & Bandettini, 2006; Power, Barnes, Snyder, Schlaggar, & Petersen, 2012; Satterthwaite et al., 2012; van Dijk, Sabuncu, & Buckner, 2012) falsely inflating predictions when head motion correlates with behavior (Shen et al., 2017). For this reason, removal of data with large motion is recommended in CPM studies (Shen et al., 2017).

Here using CPM, we investigated in 424 healthy adults whether iFC data from task‐ and rest‐fMRI sessions predict the language scores of the NIH Toolbox Cognition Battery (Gershon et al., 2014) included in the Human Connectome Project (HCP). These language measures comprise a test for vocabulary comprehension, which involved single‐word comprehension, and a test for reading recognition, which reflects reading fluency (accuracy). These language skills continue to develop as individuals learn new words throughout the lifespan (Salthouse, 1988) and can be used to estimate overall intellectual ability in healthy individuals (Beckmann, DeLuca, Devlin, & Smith, 2005). To compare the specificity of CPM to predicting language performance, we also evaluated the prediction of two nonlanguage measures: sustained attention and processing speed, which were used as control out‐of‐scan measures. For the neuroimaging datasets, we selected a language‐fMRI session and two control task‐fMRI sessions: one while performing a working memory task and the other a motor task.

We hypothesized that: (H1) the iFC models that would predict language performance (vocabulary comprehension and reading recognition) would have stronger predictions than those for the control measures (sustained attention and processing speed); (H2) the brain regions predicting language scores would include hubs located in language networks; and (H3) that the model derived from the task‐fMRI sessions would predict better language scores than the rest‐fMRI session (Greene et al., 2018; Jiang et al., 2020). As for the factors contributing to the model's prediction accuracy, we hypothesized that (H4) excluding FS from LOOCV would falsely inflate the predictions due to a high rate of false positives (Vul et al., 2009); (H5) larger sample sizes and stricter FS thresholds would increase prediction accuracy; and (H6) for larger samples (*n* > 100) prediction accuracy would not differ between LOOCV and twofold cross‐validation approaches.

## 2 MATERIALS AND METHODS

### 2.1 Subjects

We used behavioral and imaging data from the HCP (http://www.humanconnectome.org/). The 523 individuals included in the HCP 500 Subjects data release provided written informed consent as approved by the IRB at Washington University. No experimental activity with human subjects' involvement took place at the author's institutions.

The analyses were restricted to individuals for whom both phase‐encoding scans (left–right [LR]; right–left [RL]) for the rest‐fMRI session (REST), and all three task‐fMRI sessions (motor, working memory, and language) were complete and available. Ninety‐nine individuals were excluded due to incomplete image datasets, image artifacts (identified with the aid of principal component analysis), or excessive head motion (mean framewise displacement >0.4 mm). The remaining 424 participants (age: 29 ± 4 years; 243 females) were included and classified into the training sample (*n* = 212; age: 29 ± 3 years; females = 119), for the optimization of behavioral prediction models, or the test sample (*n* = 212; age: 29 ± 4 years; females = 124), for testing prediction accuracy in an independent set of subjects.

### 2.2 Language and other cognitive measures

To study the association between whole‐brain iFC and language skills we used the two language metrics obtained in the HCP sample, which corresponded to measures of vocabulary comprehension (picture vocabulary [PicVocab]), and reading recognition (oral reading recognition [ReadEng]) from the NIH toolbox (Weintraub et al., 2013). For the vocabulary comprehension test, participants were orally given a word and they had to select the picture that best matched the meaning of the word, and for the reading recognition test, participants were asked to read words as accurately as possible (Gershon et al., 2014).

We compared the accuracy of CPM for predicting language skills to that for predicting nonlanguage control measures: a sustained attention test (short Penn continuous performance [SCPT]) in which participants respond when lines form a number in the screen, and a processing speed test (pattern completion processing speed [ProcSpeed], NIH toolbox) in which participants have to discern whether two side‐by‐side pictures are the same or not.

### 2.3 fMRI tasks

To assess task‐related differences in brain–behavior associations we selected four fMRI sessions. One fMRI session was collected during the resting state (REST) and three during task performance; one while performing a language task (language task‐fMRI session) and the other two while performing working memory and motor tasks (control task‐fMRI sessions), for which procedures have been described elsewhere (Barch et al., 2013).

*Language*, LAN (Binder et al., 2011): There were four blocks of a math task interleaved with four blocks of a story task in each of two runs. The task was designed so that the length of the blocks of the math task matched those of the story task (∼30 s). During story blocks, participants listened to brief stories (five to nine sentences) followed by a two‐option forced‐choice question about the story. The participants were instructed to push a button to select either the first or the second option. The math task adaptively maintained a similar level of difficulty across participants.

*N‐back working memory*, WM (Barch et al., 2013): Four different picture categories were presented in eight separate blocks within each of two runs: faces, places, tools, and body parts. The task had a blocked design with one 0‐back (a target 2.5 s cue is presented at the start of each block; participants were instructed to press a button with the right index finger to any presentation of the target stimulus) and one 2‐back (participants were instructed to press the button whenever the current stimulus is the same as the one presented two steps back) blocks per run per category. Each block lasted 27.5 s and included ten 2.5 s trials, two of which were targets and two to three nontarget lures (repeated items in the wrong n‐back position; i.e., 1‐back or 3‐back). On each trial, the stimulus was presented for 2 s, followed by a 500 ms intertrial interval.

*Motor*, MOT (Buckner, Krienen, Castellanos, Diaz, & Yeo, 2011; Yeo et al., 2011): Visual cues requesting to tap left or right fingers, squeeze left or right toes, or move the tongue were presented to participants in a blocked paradigm. Each block lasted 12 s and was preceded by a 3 s cue. In each of two runs, there were two blocks per movement type (i.e., tongue, right hand, left hand, right foot, left foot) and three fixation blocks (15 s).

### 2.4 MRI datasets

Functional images with high spatiotemporal resolution were acquired in a 3.0 T Siemens Skyra scanner (Siemens Healthcare, Erlangen, Germany) with a 32‐channel coil using a gradient EPI sequence with multiband factor 8, TR = 720 ms, TE = 33.1 ms, flip angle 52°, 104 × 90 matrix size, 72 slices, and 2 mm isotropic voxels (Smith et al., 2013; Uğurbil et al., 2013). Scans were repeated twice using LR and RL phase encoding directions. We used the “minimal preprocessing” datasets released by the HCP, which include gradient distortion correction, rigid‐body realignment, field‐map processing, and spatial normalization to the stereotactic space of the Montreal Neurological Institute (Glasser et al., 2013). To assess the effect of motion and other noise sources on iFC, we additionally studied resting‐state datasets with and without ICA‐based X‐noiseifier, an ICA‐based automatic noise detection algorithm that can minimize various types of noise sources, including head motion (Salimi‐Khorshidi et al., 2014).

### 2.5 Image analysis

Subsequent steps were carried out using IDL (ITT Visual Information Solutions, Boulder, CO). Framewise displacements (FDs) were computed for every time point from head translations and rotations, using a 50 mm radius to convert angle rotations to displacements. Scrubbing was used to remove time points excessively contaminated with motion. Specifically, time points were excluded if the root‐mean‐square (RMS) change in blood‐oxygenation‐level‐dependent signals was >0.5% and FD > 0.5 mm (Power et al., 2012). Global signal regression (GSR) was used to remove nonneuronal sources that contribute to the global signal (Birn et al., 2006). Note that GSR also reduces the correlation magnitude between head motion and iFC (Satterthwaite et al., 2013), and strengthens the association between resting‐state iFC and behavior (Li et al., 2019). Linear regression, using the time‐varying realignment parameters, was used to minimize motion‐related fluctuations in MRI signals (Tomasi & Volkow, 2010), and to assess iFC with and without motion‐related fluctuations. Low‐pass filtering (0.10 Hz frequency cutoff) was used to attenuate high‐frequency components of the physiologic noise (Cordes et al., 2001).

Connectivity matrices, **M**, were constructed to define the iFC between regions of interest (ROIs) for each fMRI dataset and subject using the corresponding preprocessed image time series. Three different brain atlases were used as ROIs: AAL (Tzourio‐Mazoyer et al., 2002), Shen (Shen, Tokoglu, Papademetris, & Constable, 2013), and Gordon (Gordon et al., 2016), to assess the effect of brain parcellation on the accuracy of the predictions. Pearson correlation coefficients between pairs of ROIs time courses were calculated independently for LR and RL scans and normalized to z‐scores using the Fisher transformation. This resulted in 116 × 116 (AAL), 268 × 268 (Shen), and 333 × 333 (Gordon) symmetric connectivity matrices for each fMRI session and participant. The LR and RL correlation matrices corresponding to the same fMRI session were averaged to increase the signal‐to‐noise ratio in the connectivity matrices.

### 2.6 Brain–behavior prediction

**FIGURE 1:**
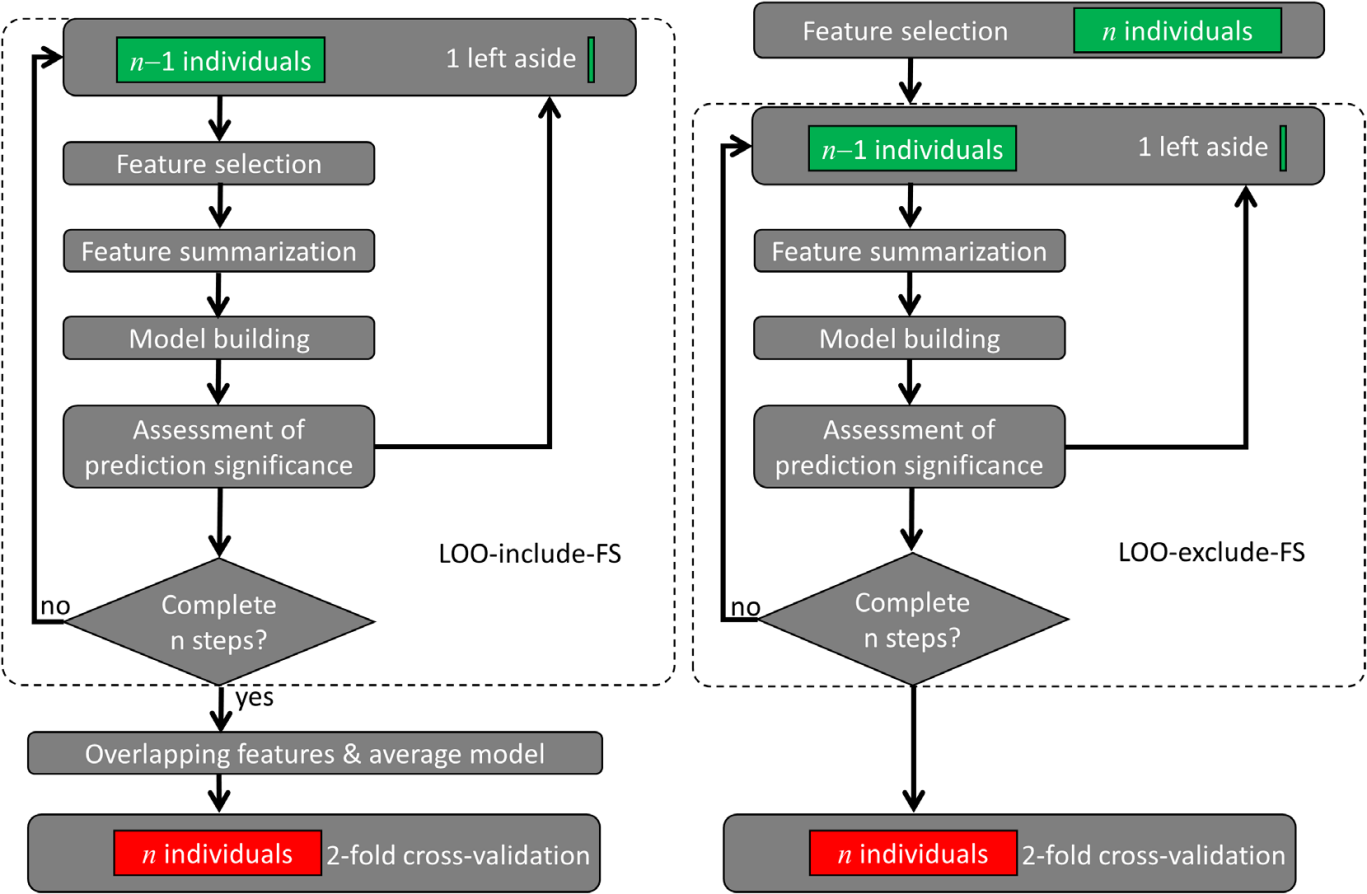
Connectome‐based predictive modeling (CPM) flowcharts. Two leave‐one‐out (LOO) cross‐validation procedures, which included (LOO‐include‐feature selection [FS]; left) or excluded (LOO‐exclude‐FS; right) FS from the LOO loop (dashed rounded rectangles). Green rectangles represent imaging and behavioral data from the training sample (*n* = 212), which were used to develop connectome‐based predictive models for predicting behavior from functional connectivity data. The red rectangle in the twofold cross‐validation step represents imaging and behavioral data from the independent test sample (*n* = 212), which was used to validate the prediction models

We used a twofold cross‐validation approach in which half of the data (training sample) was used to build the prediction models and the other half to test the optimal models in an independent sample (test sample). The optimization of the prediction models was carried out in the training sample (*n* = 212) using CPM, a data‐driven protocol for developing predictive models of brain–behavior associations based on LOOCV (Finn et al., 2015; Shen et al., 2017). To assess the effect of FS cross‐validation on the correlation between observed and predicted cognitive scores (ReadEng, PicVocab, SCPT, and ProcSpeed) across subjects (“*R*,” a benchmark metric for brain–behavior predictions), we implemented two pipelines, one in which FS was included in the LOO algorithm (LOO‐include‐FS) and the other in which it was not (LOO‐exclude‐FS; Figure 1). At each of *n* iterations in the LOO‐include‐FS pipeline, one of the *n* individuals was excluded and FS, feature summarization, and model building were carried across the remaining *n* − 1 individuals in the training sample as follows. FS: Pearson correlation was used to assess associations between a continuous cognitive score and the edges of the connectivity. Only edges that had significant positive or negative correlations with the cognitive score were identified and included in the model. Four thresholds were tested (*p* < .001, .005, .01, .05) for FS to certify that results did not depend on arbitrary threshold selection. Feature summarization: Edges with positive (negative) correlation with the cognitive score were added to compute the positive (negative) network strength, X (Y). *Model building*: a bilinear model,

(1) Ψ=a+bX+cY,

was fitted to the data across subjects in the training sample. Here, *a*, *b*, and *c* are model parameters, Ψ is the observed cognitive score, and X and Y are the positive and negative network strengths derived from the connectivity matrices. We also assessed linear models purely driven by positive or negative features by setting *c* = 0 or *b* = 0. Note that FS was excluded from LOOCV in the LOO‐exclude‐FS pipeline, as shown in Figure 1. Assessment of prediction significance: The model was then used to predict the cognitive score of the remaining individual from his/her X and Y values.

We then tested the generalizability of results to a novel group of individuals using a twofold cross‐validation approach in which the features that overlapped across the *n* LOO‐include‐FS steps, and the optimal linear and bilinear models derived from the training sample were then used to predict cognitive scores from connectivity matrices in the test sample (*n* = 212). Finally, the cross‐validation approach was completed by exchanging the roles of the training and test samples (e.g., the original test sample was used to train the model and the original training sample was used to validate the model).

### 2.7 Statistical analyses

Since training and test were independent samples, we used parametric statistics to assess the statistical significance of group differences in correlation (z‐values) between observed and predicted cognitive scores. To test for differences between two dependent correlations sharing one variable we used Williams's test (Williams, 1959) and for correlations with different variables we used Steiger's test (Steiger, 1980). Statistically significant correlations for a sample size *n* = 212 were set at *p* < .003 (*R* = .2), using Bonferroni corrections for 16 comparisons (four fMRI sessions × four scores).

### 2.8 Behavior‐head motion correlations

Pearson correlation was used to assess the associations between head motion and the language and the control measures, independently for each session, fMRI session, model, parcellation atlas, and threshold.

### 2.9 Sensitivity of *R*‐values to sample size

To assess the effect of sample size on *R*‐values we created eight sets of training and test subsamples with an increasing number of individuals (*n* = 25, 50, 75, 100, 125, 150, 175, and 200) and computed *R*‐values for each subsample for the LOO‐exclude‐FS and the LOO‐include‐FS pipelines.

### 2.10 False‐positive rate

To evaluate the false‐positive error rate, we used a “null test” in which the predicted scores did not belong to the individuals in the training sample (Ge, Tsutsumi, Aburatani, & Iwata, 2003). Thus, we assessed the rate of false positives in FS, assuming that iFC data from one individual cannot predict another individual's behavior. We assigned the cognitive scores of one of the samples (i.e., test sample) to the iFC data of the other sample (i.e., training sample). The false‐positive rate (FPR) was estimated as the ratio between the number of false‐positive edges and the total number of negatives (false positives + true negatives) for the working memory fMRI session, independently for the positive and negative network.

## 3 RESULTS

**FIGURE 2:**
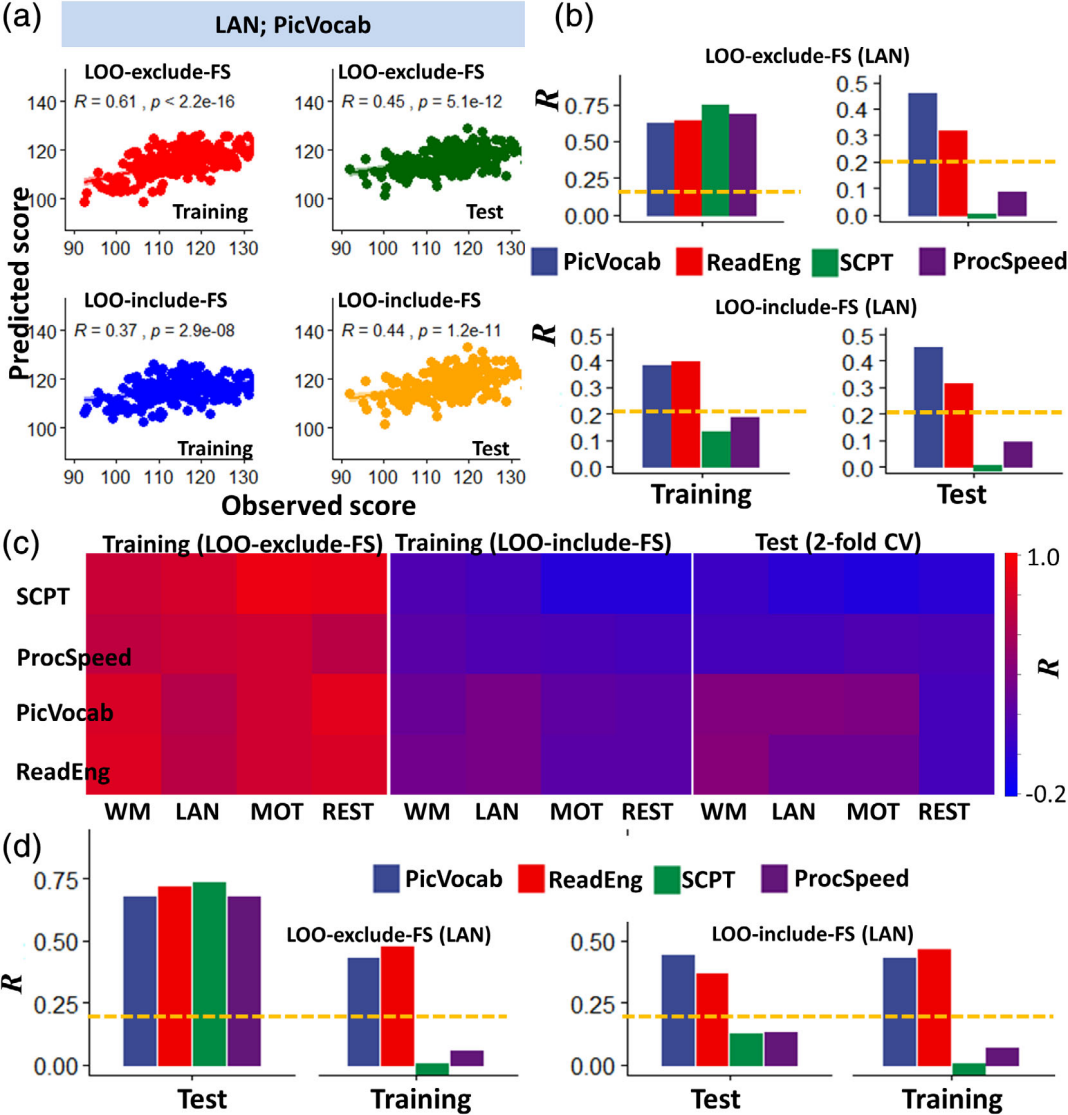
Prediction of language and other cognitive measures. (a) Linear associations between observed scores of vocabulary comprehension (PicVocab) and corresponding correlation factors (*R*) derived from language‐fMRI (LAN) data in the training sample (*n* = 212), excluding (red; leave‐one‐out [LOO]‐exclude‐feature selection [FS]) and including (blue; LOO‐include‐FS) FS in the LOO cross‐validation algorithm, and in the test sample (*n* = 212) using the optimal models (dark green and orange). (b) Pearson correlation factors between observed and predicted language and control‐task scores for the training and test samples. (c) Heat maps showing *R*‐values as a function of cognitive tests (rows) and task‐fMRI sessions (columns) for training and test (LOO‐include‐FS) samples. (d) Prediction of language and control task scores exchanging the roles of the original Training and test samples to complete the validation. Shen parcellation, language‐fMRI session, FS threshold *p* < .01, bilinear model. Abbreviations for fMRI sessions: WM, working memory; LAN, language; MOT, motor. Abbreviations for language measures: PicVocab (picture vocabulary) and ReadEng (oral reading recognition) and for control measures: ProcSpeed (processing speed) and SCPT (short Penn continuous performance). Statistical significance threshold: *p* < .05, Bonferroni corrected for 16 comparisons (dashed yellow line). *p < 2E‐16, analysis of variance (ANOVA)

Initially, we investigated whether iFC predicts behavior in the training sample, independently for three parcellation atlases (AAL, Gordon, and Shen) and three linear models at *p* < .01 or *p* < .05, the standard thresholds used in CPM studies (Rosenberg et al., 2016; Shen et al., 2017), independently for each cognitive score, fMRI session, and sample (Figure 2a–c). Finally, we validated the optimal brain–behavior models in the independent test sample (Figure 2).

### 3.1 Prediction of language and control measures

*R*‐values, computed in the training sample using the LOO‐exclude‐FS pipeline, were high for language (vocabulary comprehension: *R* = .79 ± .01; reading recognition: *R* = .82 ± .01), and control measures and for rest‐ and three task‐fMRI sessions (*p* < 3E‐14; analysis of variance [ANOVA]; Figure 2b). *R*‐values increased with the number of ROIs (*p* < 2E‐16) and did not differ between task‐ and rest‐fMRI sessions (Figure 2c) or between linear and bilinear models. For the language and control measures, and the four fMRI sessions, *R*‐values were significantly lower for the LOO‐include‐FS than for the LOO‐exclude‐FS pipeline (*p* < 2E‐16; ANOVA; Figure 2b–d). Differently, for the LOO‐include‐FS pipeline the *R*‐values were only significant for the language measures (vocabulary comprehension *R* = .39 ± .01; reading recognition: *R* = .41 ± .04) but not for the control measures (sustained attention: *R* = .13; and processing speed *R* = .18). The *R*‐values for the language measures did not differ across task‐fMRI sessions.

In the test sample, for the LOO‐exclude‐FS, the *R*‐values were significantly lower than in the training sample (*p* < 2E‐16; ANOVA; Figure 2b–d), but for the LOO‐include‐FS, the *R*‐values did not differ between the training and test samples. *R*‐values varied across cognitive measures and fMRI sessions (Figure 2b–d), being higher for language (*R* = .33 ± .13) than control (*R* = .07 ± .11) measures, and for task‐fMRI (*R* = .34 ± .25) than rest‐fMRI (*R* = .27 ± .27) (*p* < 2E‐4). There were no significant differences in *R*‐values across task‐fMRI sessions. Bilinear models predicted cognitive scores with slightly higher accuracy than linear models (*p* < .01). While *R*‐values were lower for AAL, there were no significant differences between the Shen and Gordon parcellations. *R*‐values did not differ as a function of the statistical thresholds used for FS (*p* < .05 vs. *p* < .01), consistent with previous studies (Finn et al., 2015). Thus, the most accurate predictions in the test sample were those based on the bilinear model, task‐fMRI sessions, and Shen and Gordon parcellations.

In the test sample, *R*‐values were only significant for the language measures, both for LOO‐include‐FS and LOO‐exclude‐FS estimates (*R* > .2; *p* < .05, Bonferroni corrected for 16 comparisons). For control measures, the *R*‐values were not significant (sustained attention: *R* = .01; processing speed: *R* = .16; *p* > .02, uncorrected). In the test sample, *R*‐values did not differ between LOO‐include‐FS and LOO‐exclude‐FS estimates (Figure 2c).

To ensure the robustness of our findings we exchanged the roles of the original training and test samples to complete the cross‐validation (Figure 2d). Using the LOO‐include‐FS pipeline in the original test sample, we found that the *R*‐values for the language measures remained significant.

### 3.2 Head motion

The average RMS values of realignment estimates of head motion (*d*; e.g., “absolute motion”) were larger for rest‐fMRI (*d* = 0.46 ± 0.25 mm) than task‐fMRI (*d* = 0.42 ± 0.22 mm; *p* < .0002) and did not differ between training and test samples. The average RMS values of the derivatives of head motion estimates (Δ*d*; e.g., “relative motion”) were larger for task‐fMRI (Δ*d* = 0.08 ± 0.03 mm) than rest‐fMRI (Δ*d* = 0.07 ± 0.03 mm) (*p* < 2E‐09, paired *t* test). The effects of gender and age on absolute or relative head motion were not significant.

### 3.3 Behavior versus head motion

Despite the relatively small motion excursions (*d*) and frame‐to‐frame movement (Δ*d*), there were significant correlations between language scores and Δ*d* (*r* = .22 ± .04; *p* < .001, uncorrected) but not with *d* (*r* = .09 ± .07; *p* > .2, uncorrected). After exclusion of 36 individuals with mean Δ*d* > 0.14 mm for any fMRI session, as recommended for CPM (Finn et al., 2015), the correlations of relative head motion with vocabulary comprehension remained significant (*r* = .16; *p* = .02, uncorrected) but those with reading recognition did not (*r* = .06). For the control measures, the correlations with absolute or relative head motion were not significant.

### 3.4 Effect of head motion on language prediction accuracy

**FIGURE 3:**
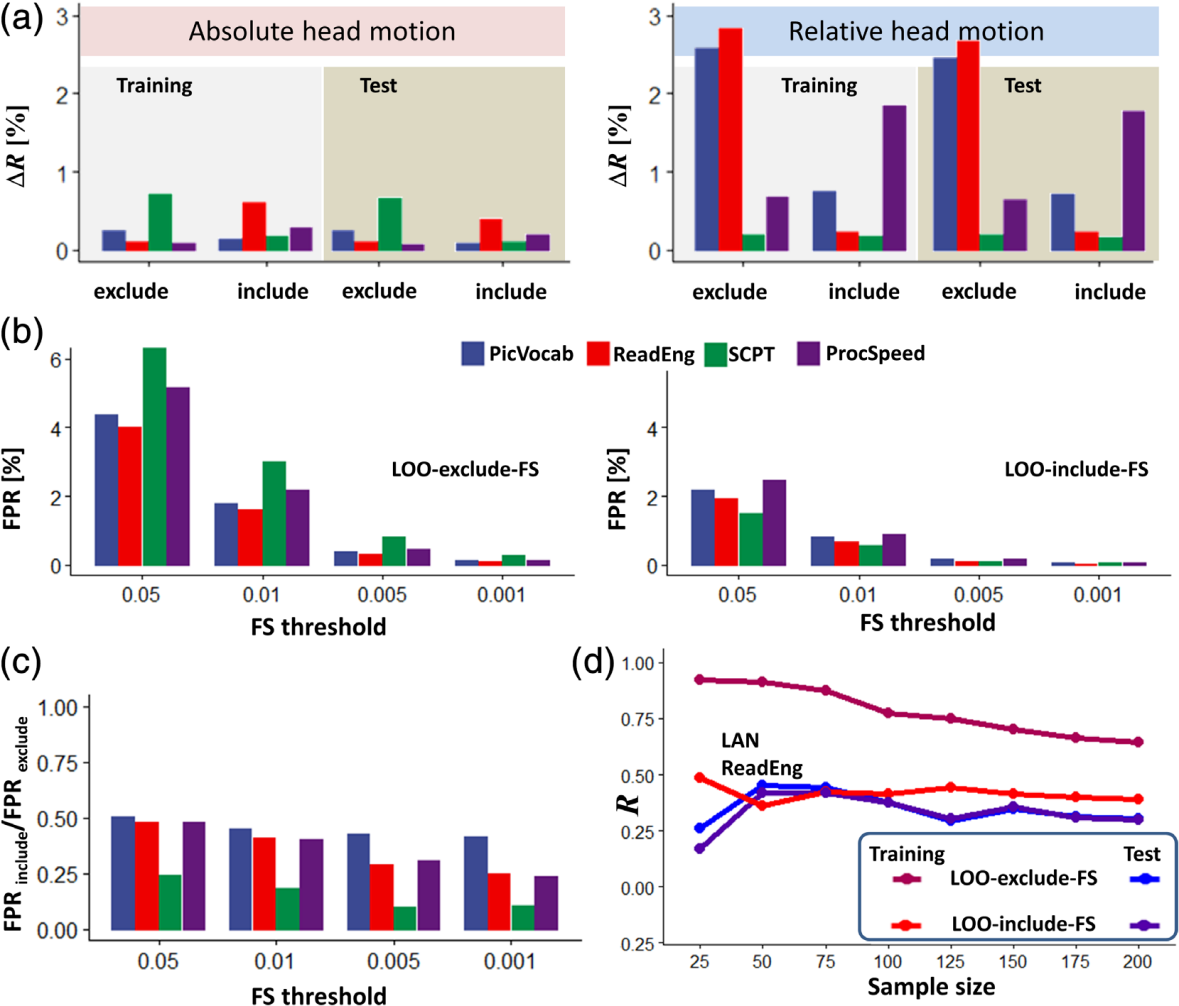
(a) Relative contributions of absolute and relative head motion to the predictions of language and the control scores. Feature selection (FS) threshold *p* < .01. (b) The false‐positive rate (FPR) in FS monotonically increased at lower statistical thresholds and was lower for the leave‐one‐out (LOO)‐include‐FS than for the LOO‐exclude‐FS pipeline. (c) FPR ratios highlighting the attenuation of false positive features when the calculation included FS in the LOO‐include‐FS loop relative to when it excluded it (LOO‐exclude‐FS). (d) Prediction of reading (ReadEng) scores from task‐fMRI for the training and test samples as a function of sample size. Bilinear model; Shen atlas; Language fMRI session. Abbreviations for fMRI sessions: WM, working memory; LAN, language; MOT, motor. Abbreviations for cognitive measures: ProcSpeed, processing speed; PicVocab, picture vocabulary; ReadEng, oral reading recognition; SCPT, short Penn continuous performance

We studied the contributions of head motion to brain‐language predictions in the full sample using motion covariates. These contributions were small, though larger for relative (0.99 ± 1.01%) than for absolute head motion (0.32 ± 0.25%) (*p* < .02; Figure 3a). For the LOO‐exclude‐FS pipeline, the contribution of Δ*d* to the prediction of language measures explained >2.5% of *R*‐values, which was significant (*p* < .05, corrected), whereas for the LOO‐include pipeline it explained <1% and was not significant. Nuisance regression of time‐varying rigid‐body translations and rotations of the head did not change significantly the *R*‐values in the training or test samples for the language or the control measures. ICA‐based denoising attenuated *R*‐values across all measures, compared to estimations without denoising, in the training but not the test sample (*p* < 5E‐05).

3.5

To assess FPR, we paired the cognitive scores of the test sample to the iFC data of the training sample, such that scores and connectivity data did not correspond to one another for any subject. Despite the pairing incongruence, the LOO‐exclude‐FS pipeline, yielded high *R*‐values across models, atlases, and thresholds (.52 < *R* < .91), reflecting its high FPR, whereas for LOO‐include‐FS *R*‐values were not significant (.01 < *R* < .07). FPR was significantly lower for LOO‐include‐FS than LOO‐exclude‐FS (*p* < 6E‐16). The high FPR in the LOO‐exclude‐FS pipeline was evident in the large fraction of edges derived from brain images in the training sample, which had significant spurious correlations with the cognitive scores from individuals in the test sample. These models, which spuriously predicted language and control measures in the test sample, failed to predict them in the training sample. We also show that for both LOO‐exclude‐FS and LOO‐include‐FS, FPR decreased with more stringent thresholds (Figure 3b). These findings highlight the importance of cross‐validation to reduce FPR and document the sensitivity of FPR to the threshold used for FS.

### 3.6 Effect of sample size

We assessed the effect of sample size on *R*‐values in three training subsamples (*n* = 25, 50, 75, 100, 125, 150, 175, and 200) and their reproducibility in eight test matched subsamples for both pipelines. With the LOO‐exclude‐FS pipeline, increased sample size decreased linearly the *R*‐values in the training subsample whereas in the test subsample it did not change them significantly (Figure 3d), reflecting the high FPR of the LOO‐exclude‐FS pipeline, which is magnified by small samples. With the LOO‐include‐FS pipeline, increased sample size did not change significantly the *R*‐values in the training or test subsamples (Figure 3d). For the test samples and except for small sample sizes (*n* < 50), *R*‐values were equivalent for LOO‐exclude‐FS and LOO‐include‐FS. For the LOO‐include‐FS pipeline and for most of the sample sizes, the *R*‐values were higher with LOOCV (training sample) than for twofold cross‐validation (test sample) for reading recognition (*p* < .05) but did not differ for vocabulary comprehension.

### 3.7 Language neuromarkers

**FIGURE 4:**
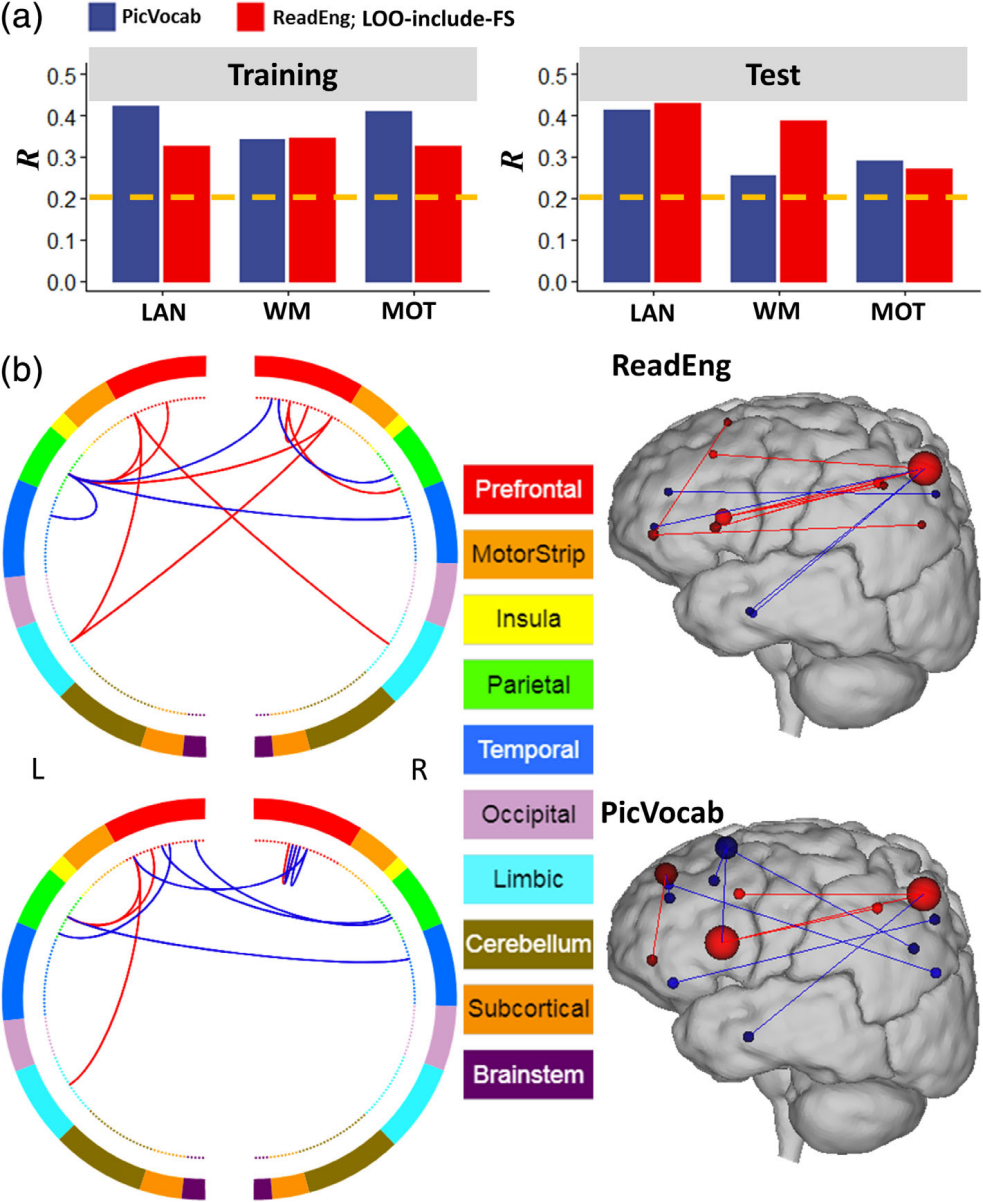
Language networks. (a) Pearson correlation values between observed and predicted cognitive scores computed from training and test subsamples with relative head motion <0.14 mm. Task‐fMRI sessions: language (LAN), working memory (WM), and motor (MOT). (b) Overlap across training and test subsamples and task‐fMRI session of the positive (red lines) and negative (blue lines) networks for reading recognition (ReadEng) and vocabulary comprehension (PicVocab) scores (left), and a glass brain plot where each node is represented as a sphere of size proportional to the number of edges of the node (right). Brain parcellation: Shen; bilinear model; training sample; LOO‐include‐FS pipeline. Figures were created with the aid of the BioImage Suite (https://bioimagesuiteweb.git)

To identify the nodes that were consistently represented across all task‐fMRI session we restricted the analysis to the training (*n* = 194) and test (*n* = 194) subsamples with low head motion (Δ*d* < 0.14 mm) and to overlapping edges emerging from the language, motor, and working‐memory task‐fMRI sessions. The networks that predicted the language scores (Figure 4a) overlapped significantly across the training and test subsamples and task‐fMRI sessions. As expected, due to the correlation between vocabulary comprehension and reading recognition (*r* = .67), these networks had similar distribution of hubs and edges (Figure 4b,c). These simpler models that comprised 12 edges (emanating from 14 hubs) for reading recognition and 11 edges (emanating from 16 hubs) for vocabulary comprehension (Table 1), accounted for 20% of the variance of the scores for the two measures (*R* = .41 ± .03, for reading recognition, and .42 ± .02 for vocabulary comprehension). The overlap of reading recognition networks comprised 12 edges whose strengths correlated positively (eight edges) or negatively (four edges) with reading recognition scores. With the aid of the BioImage Suite (https://bioimagesuiteweb.git), we observed that these hubs and edges were disproportionately located in the FPN and DMNs (Figure 4b and Table 1). Specifically, the hubs of this network were located in DMN regions (precuneus, angular, and superior frontal and medial orbitofrontal gyri, and anterior cingulum), and language areas (pars triangularis and pars orbitalis, middle and inferior temporal gyri). Similarly, the overlap of vocabulary comprehension networks comprised 11 edges whose strengths correlated positively (four edges) or negative (seven edges) with vocabulary comprehension scores that were predominantly located in FPN and DMN (Figure 4b and Table 1). The hubs and edges of this network were located in DMN regions (precuneus, angular, superior and superior medial frontal gyri, anterior cingulum and rectus), and language areas (pars triangularis and orbitalis, and middle temporal gyrus).

**TABLE 1:** Degree and MNI coordinates of hubs that predicted reading recognition (ReadEng) and vocabulary comprehension (PicVocab) scores from language‐, motor‐, and working memory‐fMRI sessions in Figure 4b. Node numbers correspond to the Shen atlas

Node	Region	BA/nucleus	Degree	MNI (mm)			Network
				x	y	z	
ReadEng							
44	Precuneus	5	6	7	−57	62	DMN
20	Pars triangularis	45	3	37	21	6	FPN
91	Precuneus	23	2	8	−40	48	DMN
155	Pars orbitalis	47	2	−32	22	6	FPN
143	Pars orbitalis	47	2	−43	47	−7	FPN
11	Pars triangularis	45/46	1	37	35	31	FPN
56	Inferior temporal	20	1	55	−8	−32	DMN
190	Middle temporal	21	1	−58	−6	−23	DMN
226	Precuneus	5	1	−9	−43	50	DMN
182	Angular	39	1	−42	−66	42	DMN
178	Precuneus	7	1	−10	−66	55	DMN
148	Superior frontal	9	1	−11	34	52	DMN
140	Anterior cingulum	32	1	−6	48	12	DMN
138	Medial orbitofrontal	10	1	−7	48	−6	DMN
PicVocab							
44	Precuneus	5	3	7	−57	62	DMN
20	Pars triangularis	45	3	37	21	6	FPN
148	Superior frontal	9	2	−11	34	52	DMN
145	Superior med frontal	10	2	−10	56	30	DMN
190	Middle temporal	21	1	−58	−6	−23	DMN
143	Pars orbitalis	47	1	−43	47	−7	FPN
146	Middle frontal	9	1	−27	34	36	FPN
144	Middle frontal	46	1	−29	50	22	FPN
178	Precuneus	7	1	−10	−66	55	DMN
91	Precuneus	23	1	8	−40	48	DMN
49	Angular	39	1	41	−75	28	DMN
177	Angular	7	1	−28	−62	40	DMN
15	Anterior cingulum	24	1	7	21	32	DMN
12	Superior frontal	9	1	14	37	49	DMN
10	Anterior cingulum	32	1	8	53	24	DMN
3	Rectus	11	1	5	35	−17	DMN

Abbreviations: DMN, default‐mode network; FPN, frontoparietal network; MNI, Montreal Neurological Institute.

## 4 DISCUSSION

Using imaging data from four different fMRI sessions, we document moderate but significant predictability for vocabulary comprehension and reading recognition from fMRI data in healthy controls that was reproducible in an independent sample, corroborating hypothesis H1. Specifically, iFC data from language‐, motor‐, or working memory‐fMRI sessions explained >20% of the variance of the language measures, whereas the rest‐fMRI session explained <14% of the variance. In contrast iFC data from task‐ or rest‐fMRI sessions explained <3% of the variance of the control measures.

Jiang et al. recently reported, using partial least square regression analyses on a similar sample of the HCP, a predictive model that integrated the networks derived from six fMRI sessions that maximized the prediction accuracy and that corresponded for reading recognition to *R* = .498 and for vocabulary comprehension to *R* = .503 and explained 25% of the variance. Instead, we report on a much simpler model derived from the edges that overlapped across the three task‐fMRI conditions that explained 20% of the variance of the language scores and that relied only on 12 and 11 edges for the reading recognition and the vocabulary comprehension, respectively. Though the integrated model accounted for a larger portion of the variance than our overlapping model (25% vs. 20%) it relied on >400 edges in contrast to reliance on 12 edges for reading recognition and 11 edges for vocabulary comprehension.

The hubs that predicted language measures (14 for reading recognition and 16 for vocabulary comprehension) were prominently located in language areas encompassing FPN and DMN, consistent with connectivity models predicting language performance (Jiang et al., 2020) and with reports of FPN's engagement in language processing (Geranmayeh, Wise, & Leech, 2014; Lohmann et al., 2010; Tomasi & Volkow, 2012), and of DMN's engagement in language comprehension (Tesink et al., 2009).

Notably, the network hubs that predicted reading recognition included the following language areas: Broca's area (posterior inferior frontal gyrus; BA 45 and 44), which is associated with language production (Sahin, Pinker, Cash, Schomer, & Halgren, 2009) and comprehension (Skipper, Goldin‐Meadow, Nusbaum, & Small, 2007); pars orbitalis (BA 47), which is implicated in semantic and phonological processing of language (Ardila, Bernal, & Rosselli, 2016); the angular gyrus, which is involved in speaking and writing (Brownsett & Wise, 2010), including findings of increased functional connectivity between the angular gyrus and Broca's and Wernicke's areas during reading (Segal & Petrides, 2013); and the anterior temporal cortex (BAs 20 and 21), which is implicated in semantic processing (Humphries, Love, Swinney, & Hickok, 2005; Visser, Jefferies, & Lambon Ralph, 2010). Similarly, the network hubs that predicted vocabulary comprehension included the same language areas, corroborating hypothesis H2.

The right precuneus was a prominent DMN hub in the networks that predicted language scores. Early on, PET imaging studies had identified the relevance of the precuneus for language comprehension (Bottini et al., 1994; Musso et al., 1999) and subsequent resting‐state fMRI studies showed anticorrelated functional connectivity between precuneus and Broca's and Wernicke's areas (Tomasi & Volkow, 2012). In task‐fMRI studies, activation of the precuneus was interpreted to reflect the attentional component when performing language tasks (Cohen et al., 2002; McDermott, Petersen, Watson, & Ojemann, 2003).

Our findings have potential clinical relevance in that by identifying the main hubs in networks that predict language performance, namely pars triangularis, pars orbitalis, anterior temporal cortex, precuneus, and angular gyrus, they provide with potential targets for neuromodulation using technologies such as rTMS (Naeser et al., 2013) or tDCS (Fridriksson et al., 2018). Future studies are needed to assess if neuromodulation of these hubs improves language performance in the elderly and to assess its value in the rehabilitation of patients with language disorders. Mapping of key hubs that influence language performance and their functional connections is also relevant for presurgical planning to minimize the likelihood of surgery‐associated language impairments.

Surprisingly, the overlapping edges in networks predicting language scores, did not include the left fusiform gyrus, which is engaged in word reading (McCandliss, Cohen, & Dehaene, 2003) and for which its reactivity to letters has been shown to predict reading ability in children and its hypoactivation associated with reading impairments (Centanni et al., 2019). The reason(s) why the fusiform gyrus was not represented among the overlapping edges from the language‐predicting networks is unclear, but it could reflect the different task‐fMRI sessions used to identify overlap. In this respect, only one of the fMRI tasks was a language task that required participants to listen to a story and it is possible that additional language task‐fMRI sessions might have given more prominence to this region. Also, even though the simplified network based on the overlapping edges across the networks predicted 20% of the variance in the language scores, 80% of the variance was unexplained, which highlights the need of further research to maximize the sensitivity of task‐fMRI to construct predictive models of language performance in healthy adults.

### 4.1 Task versus rest

Task‐free iFC can be used to assess language networks without requiring participants to perform cognitive tasks (Battistella et al., 2020; Tomasi & Volkow, 2012). However, previous CPM studies have documented higher *R*‐values for task‐fMRI than for rest‐fMRI using LOOCV (Greene et al., 2018; Jiang et al., 2020), which is consistent with our data. Specifically, compared to rest‐fMRI, task‐fMRI improved prediction accuracy for the language measures, corroborating H3.

### 4.2 Cross‐validation and sample size

The *R*‐values for the language measures with the LOO‐include‐FS pipeline were validated using LOOCV in the training sample and twofold cross‐validation in the test sample. Overall, the reproducibility of the *R*‐values for the LOO‐include‐FS pipeline was good. In contrast, *R*‐values with the LOO‐exclude‐FS pipeline in the training sample were high (*R* ∼ .75), but their reproducibility in the test sample was very poor. These findings corroborate working hypothesis H4 and highlight the importance of including FS within the LOOCV loop. Since the predictability of behavior from imaging data is low (less than 20% of variance in the case of language measures for this study), and the sample sizes in many imaging studies are small (*n* < 100), reproducibility of the findings in independent samples is needed to ensure the validity of the results (Scheinost et al., 2019).

### 4.3 False positives

Brain–behavior predictions with the LOO‐include‐FS pipeline had low/moderate *R*‐values, which is consistent with prior studies (Beaty et al., 2018; Bellucci et al., 2019; Emerson et al., 2017; Fountain‐Zaragoza et al., 2019; Greene et al., 2018; Lin et al., 2018; Liu et al., 2018; Rosenberg et al., 2016; Scheinost et al., 2019). The high *R*‐values obtained with the LOO‐exclude‐FS pipeline were due to the marked inflation of spurious correlations due to overfitting. Indeed, the LOO‐exclude‐FS pipeline increased 100% the FPR and inflated *R*‐values by a factor of 3. Cross‐validation in an independent sample protected against the high FPR with the LOO‐exclude‐FS pipeline. We also show that larger samples and lower FS threshold decrease FPR, corroborating hypothesis H5. Our findings highlight the inadequacy of the LOO‐exclude‐FS pipeline for CPM studies even for those with large samples sizes, particularly if they do not corroborate with cross‐validation. For the LOO‐include‐FS pipeline with samples *n* > 50, the *R*‐values were similar for the LOO and twofold cross‐validation approaches, corroborating hypothesis H6.

### 4.4 Head motion

The removal of individuals with large frame‐to‐frame head motion could introduce sampling bias (Wylie, Genova, DeLuca, Chiaravalloti, & Sumowski, 2014). In this study, however, *R*‐values in the sample with moderate motion (Δ*d* < 0.4 mm) did not differ from those in the subsample with low motion (Δ*d* < 0.14 mm). Thus, removal of data with excess motion that allowed us to eliminate significant correlations between behavior and head motion did not cause significant sampling bias in our study. It is also believed that significant correlations between behavior and head motion can spuriously increase *R*‐values (Rosenberg et al., 2016; Shen et al., 2017). However, head motion contributed minimally to predictions of vocabulary comprehension and reading recognition scores (<1%).

### 4.5 CPM: Language versus control measures

With cross‐validation, vocabulary comprehension and reading recognition had significantly higher *R*‐values than sustained attention and processing speed. Supporting our hypothesis (H1), this suggests that functional connectivity measures, whether at rest or during task conditions, are more sensitive to language processing skills than those related to sustained attention and speed processing at least as it relates to the tests included in the NIH toolbox.

In summary, we show that functional connectivity strength is a reproducible predictor of language measures in healthy controls and that relatively few edges can predict 20% of the variance for reading recognition (12 edges) and for vocabulary comprehension (11 edges). Furthermore, this study highlights the importance of twofold cross‐validation in brain–behavior prediction studies to ensure the robustness of the associations and documents that inclusion of FS within the LOOCV loop of CPM is crucial to mitigate the high rate of false positives in brain–behavior correlations.

## Data Availability

We used behavioral and imaging data from the Human Connectome Project (HCP; http://www.humanconnectome.org/).
